# Immunostick Test for Detecting ζ-Globin Chains and Screening of the Southeast Asian α-Thalassemia 1 Deletion

**DOI:** 10.1186/s12575-019-0104-2

**Published:** 2019-08-01

**Authors:** Supansa Pata, Matawee Pongpaiboon, Witida Laopajon, Thongperm Munkongdee, Kittiphong Paiboonsukwong, Sakorn Pornpresert, Suthat Fucharoen, Watchara Kasinrerk

**Affiliations:** 1 Division of Clinical Immunology, Department of Medical Technology, Faculty of Associated Medical Sciences, Chiang Mai University, Chiang Mai, 50200 Thailand; 2 Biomedical Technology Research Center, National Center for Genetic Engineering and Biotechnology National Science and Technology Development Agency at the Faculty of Associated Medical Sciences, Chiang Mai University, Chiang Mai, 50200 Thailand; 3 Thalassemia Research Center, Institute of Molecular Biosciences, Mahidol University, Nakorn Pathom, 73170 Thailand; 4 Division of Clinical Microscopy, Department of Medical Technology, Faculty of Associated Medical Sciences, Chiang Mai University, Chiang Mai, 50200 Thailand

**Keywords:** Immunostick test, ζ-Globin chain, (−-(SEA)) α-thalassemia 1, Thalassemia screening test

## Abstract

**Background:**

Couples who carry α-thalassemia-1 deletion are at 25% risk of having a fetus with hemoglobin Bart’s hydrops fetalis. Southeast Asian deletion (−-(SEA)) is the most common type of α-thalassemia 1 among Southeast Asian populations. Thus, identification of the (−-(SEA)) α-thalassemia 1 carrier is necessary for controlling severe α-thalassemia in Southeast Asian countries.

**Results:**

Using our generated anti ζ-globin chain monoclonal antibodies (mAbs) clones PL2 and PL3, a simple immunostick test for detecting ζ-globin chain presence in whole blood lysates was developed. The procedure of the developed immunostick test was as follows. The immunostick paddles were coated with 50 μg/mL of mAb PL2 as capture mAb, or other control antibodies. The coated immunostick was dipped into cocktail containing tested hemolysate at dilution of 1:500, 0.25 μg/mL biotin-labeled mAb PL3 and horseradish peroxidase-conjugated streptavidin at dilution of 1:1000. The immunostick was then dipped in precipitating substrate and the presence of ζ-globin chain in the tested sample was observed by the naked eye. Upon validation of the developed immunostick test with various types of thalassemia and normal subjects, 100% sensitivity and 82% specificity for detection of the (−-(SEA)) α-thalassemia-1 carriers were achieved. The mAb pre-coated immunostick can be stored at room temperature for at least 20 weeks.

**Conclusion:**

In this study, a novel simple immunostick test for the screening of (−-(SEA)) α-thalassemia 1 carriers was presented. The developed immunostick test, within a single test, contains both positive and negative internal procedural controls.

## Introduction

Hemoglobin (Hb) Bart’s hydrops fetalis is the most severe type of thalassemia, in which the fetus suffers from severe anemia, hypoxia and mortality in utero. α-thalassemia 1 is a thalassemia caused by deletion of two α-globin genes in *cis.* Married couples who carry the α-thalassemia 1 trait have a 25% risk of Hb Bart’s hydrops fetalis in each pregnancy as per the absence of α-globin gene [1–3]. With improper care, mothers carrying a hydropic fetus can develop severe preeclampsia, placental abruption and antepartum hemorrhage [4, 5]. In Southeast Asian populations, within the α-thalassemia 1 trait, the Southeast Asian (SEA) type deletion, named SEA type α-thalassemia 1, is the most common genotype [6–10]. To prevent homozygous α-thalassemia 1 (Hb Bart’s hydrops fetalis) in these populations, determination (−-(SEA)) α-thalassemia-1 trait and counseling are, therefore, necessary.

Currently, several screening tests for diagnosis of α-thalassemia 1 are available. Complete blood count, red blood cell indices and osmotic fragility test are the simple tests commonly used; however, sensitivity and specificity are low [11]. Genotyping using polymerase chain reaction (PCR) is the standard technique to detect α-thalassemia 1 carriers with high sensitivity [11, 12]. However, complicated procedures, time consumption and the requirement for a well-equipped laboratory and expert staff make the genotyping methods unaffordable, especially in resource-limited countries. In (−-(SEA)) α-thalassemia-1 trait, ζ-globin chains have been reported to be present in red blood cells [13–16]. Consequently, immunoassays have been developed for the detection of ζ-globin chains in blood samples and applied for screening of (−-(SEA)) α-thalassemia-1 carriers [13, 14, 16–20]. Previously, we have developed the modified ELISA for the detection of ζ-globin chains using our generated monoclonal antibodies (mAbs) against ζ-globin chains, named mAb PL2 and mAb PL3 [19]. However, the developed ELISA was not appropriate for routine screening of thalassemia.

In this study, we further established a simple immunostick test for ζ-globin chain detection. The developed immunostick test could be used for screening (−-(SEA)) α-thalassemia-1 carriers with a very high sensitivity. This method is simple, easily performed and the results could be determined by the naked eye. This test is therefore suitable for screening in mass population as well as individual testing, especially in remote areas.

## Results

### Immunostick Test for Detection of ζ-Globin Chain in Blood Sample

**Fig. 1:**
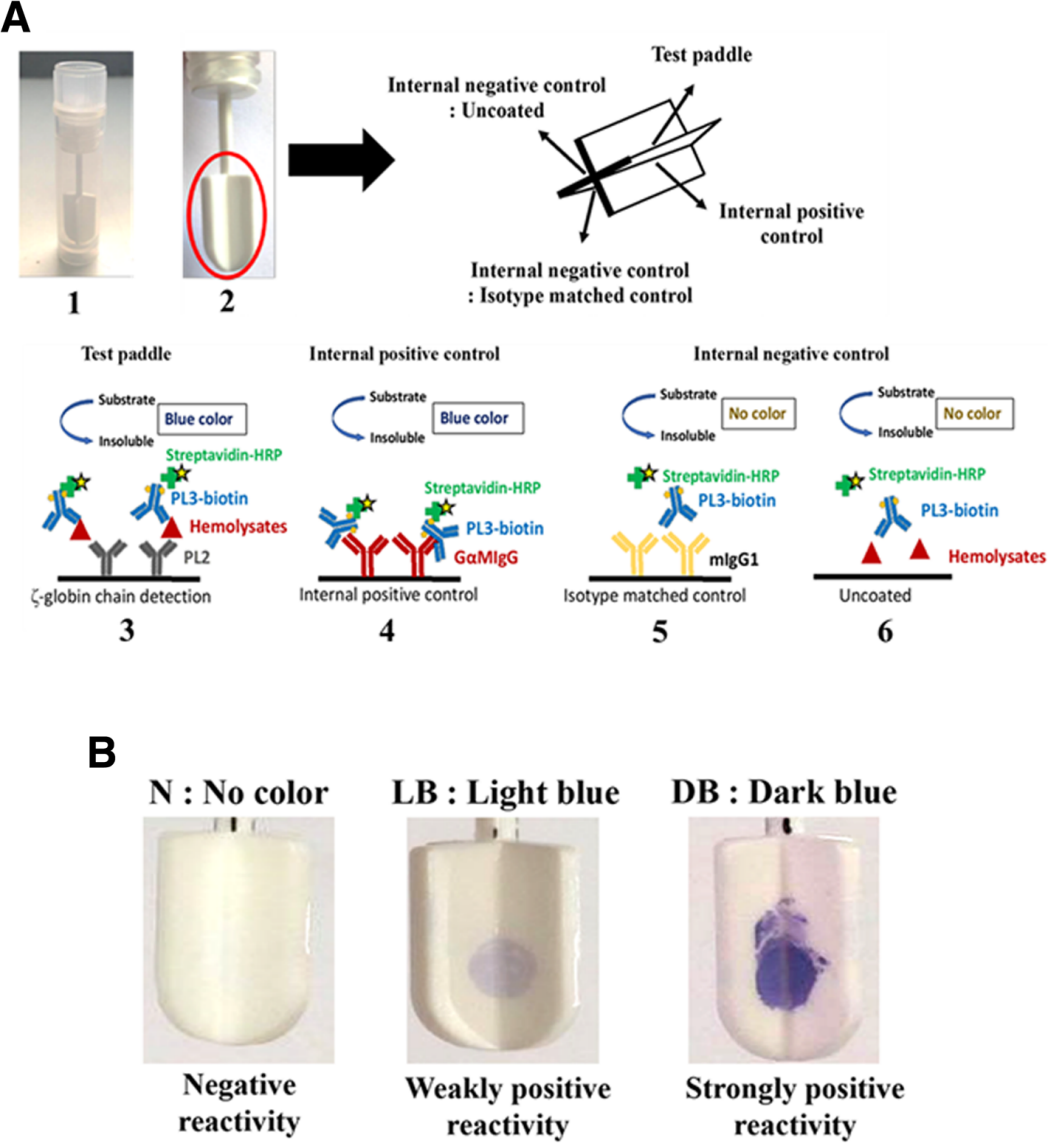
Schematic of sandwich type immunostick test. **a** The model of double antibody sandwich immunostick and internal control design. Immunostick was obtained as individual tube (1). Each stick consists of four paddles (as indicated by red circle) and each paddle was designed as test, positive control, isotype-matched mAb control and uncoated negative control, as indicated (2). The test paddle was coated with mAb PL2 to capture ζ-globin chain (3). Internal positive control was coated with goat anti-mouse immunoglobulins antibody (4). Internal negative control composes of isotype-matched mAb control and uncoated negative control. The isotype-matched mAb control was coated with mouse IgG1 mAb (5). The uncoated negative control was the paddle that left as uncoated paddle (6). Biotinylated mAb PL3 was used to detect captured ζ-globin chain and HRP-conjugated streptavidin (Streptavidin-HRP) was used to monitor the antigen-antibody complexes. Finally, sticks were dipped into precipitating TMB substrate. Positive reaction (color spot) was observed with the naked eye. **b** For determining the presence of ζ-globin chain, the color spots were graded as no color (N): negative reactivity; light blue (LB): weakly positive reactivity; and dark blue (DB): strongly positive reactivity

Immunostick test is a solid phase immunoassay. In this study, double antibody sandwich immunostick assay was developed for the detection of ζ-globin chains in blood samples. As we have previously generated two anti ζ-globin chain mAbs [19], the mAbs were incorporated in the immunostick test. An immunostick contains four paddles with high protein binding properties. We, therefore, designed the immunostick test as shown in Fig. 1. Within each immunostick test (4 paddles), test, positive control, uncoated negative control and isotype-matched mAb control were included and simultaneously verified in the same sample (Fig. 1a). For determining the presence of ζ-globin chains in samples, the spotted colors on each stick paddle were observed with the naked eye and graded according to the color intensity (no color (N): no reactivity; Light blue (LB): weakly positive reactivity; dark blue (DB): strongly positive reactivity) (Fig. 1b).

### Determination of the Optimal Concentration of Capture mAb on the Immunostick Test

In our immunostick test, anti ζ-globin chain mAb clone PL-2 was used as the capture mAb (Fig. 1a). The optimal concentration of capture mAb PL2 was then determined by block titration. As shown in Table 1, 50 and 100 μg/mL of mAb PL2 coated paddles showed strongly positive reactivity (dark blue dot) with hemolysate containing ζ-globin chains whereas weakly positive reactivity (light blue dot) was observed on paddle coated with 20 μg/mL of mAb PL2. With normal subject hemolysate, 100 μg/mL of mAb PL2 coated paddle, however, showed false positive reactivity (light blue dot), while paddle coated with 20 and 50 μg/mL of mAb PL2 showed negative reactivity. From these results, 50 μg/mL of mAb PL2 was selected as the optimal concentration for using as capture mAb PL2 on the stick.

**Table 1:** Titration of the concentration of captured anti ζ-globin chain mAb clone PL2

Hemolysates	PL2 mAb (μg/mL)			Isotype matched mAb (μg/mL)
	20	50	100	100
Hb Bart’s hydrops fetalis (containing 5 μg/mL of ζ-globin)	LB^a^	DB^b^	DB	N^c^
Normal	N	N	LB	N

^a^ Light blue: Weakly positive reactivity

^b^ Dark blue: Strongly positive reactivity

^c^ No color: Negative reactivity

### Determination of Optimal Dilution of Hemolysate for Use in Immunostick Test

In our experiments, we found that if undiluted hemolysate was used, high assay background was occurred with the developed immunostick test. Therefore, an optimal dilution of hemolysate used needed to be determined. As shown in Table 2, within 10 non-SEA type α-thalassemia 1 hemolysates, while some hemolysates (samples N1, N3, N4, N5, N7, N9) at 1:250 dilution showed false positive, all tested hemolysate at dilutions of 1:500, 1:1000 and 1:2000 was negative with the test and negative control paddles. The positive control paddles were always positive. Hemolysate at dilution of 1:500 was, therefore, selected as the optimal dilution for use in immunostick test.

**Table 2:** Determination of optimal dilution of hemolysates

Sample number	Hemolysates of non-SEA type α-thalassemia 1											
	Dilution 1:250				Dilution 1:500				Dilution 1:1000			
	Test paddle	Internal control paddles			Test paddle	Internal control paddles			Test paddle	Internal control paddles		
		Positive control	Negative control			Positive control	Negative control			Positive control	Negative control	
			Isotype matched control	Uncoat control			Isotype matched control	Uncoat control			Isotype matched control	Uncoat control
N1	LB^a^	DB^b^	LB	N^c^	N	DB	N	N	N	DB	N	N
N2	N	DB	N	N	N	DB	N	N	N	DB	N	N
N3	LB	DB	LB	N	N	DB	N	N	N	DB	N	N
N4	LB	DB	LB	N	N	DB	N	N	N	DB	N	N
N5	LB	DB	LB	N	N	DB	N	N	N	DB	N	N
N6	N	DB	N	N	N	DB	N	N	N	DB	N	N
N7	LB	DB	N	N	N	DB	N	N	N	DB	N	N
N8	N	DB	N	N	N	DB	N	N	N	DB	N	N
N9	LB	DB	LB	N	N	DB	N	N	N	DB	N	N
N10	N	DB	N	N	N	DB	N	N	N	DB	N	N

^a^Light blue: Weakly positive reactivity

^b^Dark blue: Strong positive reactivity

^c^No color: Negative reactivity

### Development of Simple immunostick test

**Fig. 2:**
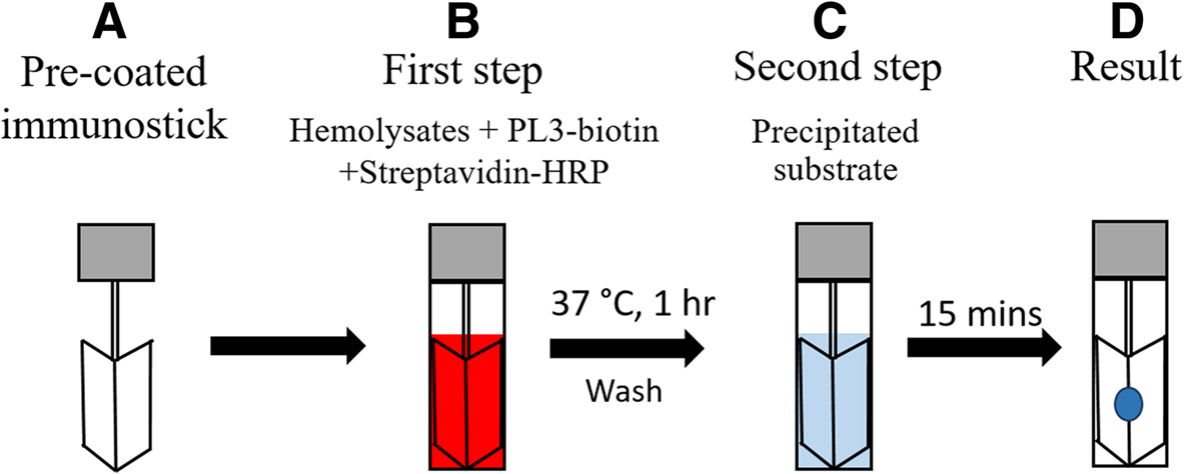
Diagram of simple immunostick test for the screening of SEA type deletion α-thalassemia 1. **(**A) Pre-coated immunostick containing 4 paddles was prepared as Fig. 1a (test paddle: mAb PL2; positive control paddle: goat anti-mouse immunoglobulins antibody; isotype-matched mAb control paddle: mouse IgG1 mAb; and negative control paddle: left as uncoated). (B) For the first step, immersion of the pre-coated immunostick into cocktail containing tested hemolysate, detecting mAb (biotinylated-mAb PL3; PL3-biotin), and HRP-conjugated streptavidin (streptavidin-HRP) at 37 °C for 1 h. (C) For second step, the immunostick was dipped into precipitating TMB substrate. (D) The positive reaction (presence of ζ-globin chain) was seen as a blue color spot on the stick paddle. A brief washing between first step and second step was done by dipping stick in 0.05% Tween-PBS and agitating for 5 s

In order to shorten the processing step and incubation time, a simple immunostick test was further developed. The principle of the test was shown in Fig. 2. The processing steps were shortened by dipping the pre-coated immunostick into the cocktail solution containing tested hemolysate, detecting mAb (biotinylated mAb PL3) and HRP-conjugated streptavidin. After incubation, the stick was then dipped into precipitating TMB substrate. The blue color spots appearing on the stick paddles were observed with the naked eye.

Previously, we demonstrated that the SEA type α-thalassemia 1 hemolysate contained ζ-globin chain ranges between 6.43 and 915.36 μg/mL [19]. Thus, the estimated maximum concentration of ζ-globin chain in hemolysate at dilution of 1:500 should be 1.8 μg/mL. To avoid postzone phenomenon, we therefore tested 9 mixture types of cocktail solution which contained biotinylated detecting mAb PL3 of 0.125, 0.25 and 0.5 μg/mL and HRP-conjugated streptavidin at 1:500, 1:1000 and 1:2000 for detecting 5 μg/mL of ζ-globin chains.

As shown in Table 3, hemolysate containing 5 μg/mL ζ-globin chains showed positive reactivity (dark blue dot) on the test paddles in all tested conditions. When using the cocktail solution containing streptavidin-HRP at dilution of 1:500, however, hemolysate containing ζ-globin chain not only showed a strong positive on the test paddles but also showed some background (light blue dot) on isotype-matched mAb control paddles. Furthermore, with normal hemolysate (no ζ-globin chain), the background was also observed on test paddles and isotype-matched control paddles. These results indicated that streptavidin-HRP at dilution of 1:500 was not appropriate for the immunostick test.

**Table 3:** Determination of the appropriate cocktail solution containing hemolysates and biotin-PL3 and HRP-streptavidin

Cocktail mixtures		Hb Bart’s hydrops fetalis hemolysates (containing 5 μg/mL of ζ-globin)				Normal hemolysates dilution 1:500			
		Test paddle	Internal control paddles			Test paddle	Internal control paddles		
PL3-biotin (μg/mL)	Streptavidin-HRP (dilution)		Positive control	Negative control			Positive control	Negative control	
				Isotype matched control	Uncoat cpntrol			Isotype matched control	Uncoat control
0.125	1:500	DB^b^	DB	LB^a^	N^c^	LB	DB	LB	N
0.250		DB	DB	LB	N	LB	DB	LB	N
0.500		DB	DB	LB	N	LB	DB	LB	N
0.125	1:1000	DB	DB	N	N	N	DB	N	N
0.250		DB	DB	N	N	N	DB	N	N
0.500		DB	DB	LB	N	LB	DB	LB	N
0.125	1:2000	LB	DB	N	N	N	DB	N	N
0.250		LB	DB	N	N	N	DB	N	N
0.500		LB	DB	N	N	N	DB	N	N

^a^Light blue: Weakly positive reactivity

^b^Dark blue: Strongly positive reactivity

^c^No color: Negative reactivity

When using cocktail solution containing streptavidin-HRP at dilution of 1:1000, the background on isotype-matched control mAb paddles was observed only under the condition of using mAb PL3 0.5 μg/mL. Meanwhile, hemolysate containing ζ-globin chain showed positive reactivity on the test paddle with no background on isotype-matched mAb control paddles under the condition of using 0.125 and 0.25 μg/mL of mAb PL3.

In the cocktail solution containing HRP labelled streptavidin at dilution of 1:2000, hemolysate containing ζ-globin chain showed positive reactivity on the test paddles with no background on isotype-matched mAb control paddles. The apparent blue color dot of the test paddle was, however, weak (light blue dot).

As per the obtained results, we selected 0.25 μg/mL of biotinylated PL3 and HRP-conjugated streptavidin at dilution of 1:1000 as optimal conditions for the cocktail solution.

Taken together, the optimal condition for a simple immunostick test was using of 50 μg/mL capture mAb PL2 for pre-coated immunostick. For the cocktail solution, 0.25 μg/mL of detecting biotinylated mAb PL3 mixed with HRP-conjugated streptavidin at dilution of 1:1000 in detecting ζ-globin chain of 1:500 tested hemolysate was the optimal condition.

**Fig. 3:**
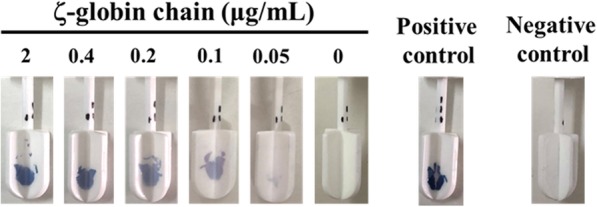
Validation of simple immunostick test with various concentrations of ζ-globin chain. Sticks were coated with anti ζ-globin chain mAb, goat anti-mouse immunoglobulins antibody (positive control) or isotype-matched mAb control (negative control). Then, the immunosticks were blocked with 2% skimmed milk in PBS. Anti ζ-globin mAb coated immunosticks were dipped in the hemolysate containing various concentrations of ζ-globin chain as indicated. The positive and negative control immunosticks were dipped in the hemolysate containing 2 μg/mL ζ-globin. Color spots were developed as described in Fig. 2

The immunostick test was then validated with hemolysates containing various concentrations of ζ-globin chain. As shown in Fig. 3, the intensity of the blue color spots appearing on the stick paddles were positively correlated with the concentration of ζ-globin chain in the sample. The detection limit of the immunostick test was 0.1 μg/mL.

### Validation of the developed simple immunostick test for screening of SEA type deletion α-thalassemia 1 carriers

To evaluate the developed simple immunostick test, 53 SEA type α-thalassemia 1 samples and 95 non-SEA type α-thalassemia 1 samples were tested. The results were summarized in Table 4. The sensitivity, specificity, positive predictive value (PPV) and negative predictive value (NPV) of the immunostick test for SEA type α-thalassemia 1 detection were 100, 82, 76 and 100%, respectively.

**Table 4:** Validation of developed immunostick test for screening of α-thalassemia 1 SEA type deletion carriers

Immunostick test for ζ-globin chain	PCR analysis for SEA 훼-thalassemia 1		Total
	Positive	Negative	
Positive	53	17	70
Negative	0	78	78
Total	53	95	148

Sensitivity (53/53) × 100 = 100%

Specificity (78/95) × 100 = 82%

Positive predictive value (53/70) × 100 = 76%

Negative predictive value (78/78) × 100 = 100%

### Stability of the Pre-Coated Immunostick Test

We further prepared the pre-coated immunosticks by coating 50 μg/mL of mAb PL2, 0.5 μg/mL of goat anti-mouse IgG antibody and 50 μg/mL of mouse IgG1 mAb on stick paddles. Then, the sticks were covered with 10% glucose and dried. The pre-coated immunosticks were stored at room temperature for 20 weeks. The stability of the pre-coated immunosticks was tested with hemolysate containing 0, 0.25 and 0.5 μg/mL ζ-globin chains. It was found that pre-coated immunosticks remained stable for at least 20 weeks (Table 5).

**Table 5:** Stability of pre-coated immunostick test

Time of storage	Normal hemolysates dilution 1:500				Hb Bart’s hydrops fetalis hemolysates							
					containing 0.25 μg/mL ζ-globin				containing 0.5 μg/mL ζ-globin			
	Test paddle	Internal control paddles			Test paddle	Internal control paddles			Test paddle	Internal control paddles		
		Positive control	Negative control			Positive control	Negative control			Positive control	Negative control	
			Isotype matched control	Uncoat control			Isotype matched control	Uncoat cotrol			Isotype matched control	Uncoat control
Day 1	N^c^	DB^b^	N	N	LB^a^	DB	N	N	DB	DB	N	N
Week 1	N	DB	N	N	LB	DB	N	N	DB	DB	N	N
Week 5	N	DB	N	N	LB	DB	N	N	DB	DB	N	N
Week 10	N	DB	N	N	LB	DB	N	N	DB	DB	N	N
Week 15	N	DB	N	N	LB	DB	N	N	DB	DB	N	N
Week 20	N	DB	N	N	LB	DB	N	N	DB	DB	N	N

^a^Light blue: Weakly positive reactivity

^b^Dark blue: Strongly positive reactivity

^c^No color: Negative reactivity

## Discussion

Diagnosis of α-thalassemia 1 carriers is an important step for the prevention of Hb Bart’s hydrops fetalis, which is the most severe thalassemia disease. Several methods, including ζ-globin chain detection, have been developed for α-thalassemia-1 screening [13, 14, 16–20]. Previously, using our generated anti-ζ-globin chain mAbs, an ELISA for detecting ζ-globin chain had been developed [19]. The ELISA in microplate platform, however, has several drawbacks, including time consumption, complicated procedures, the requirement of sophisticated laboratory instrumentation with well-trained technicians and unsuitability for conducting individual tests [13, 14, 16–21]. In this study, we modified our ELISA to an immunostick test platform. Based on the use of stick-type solid support and precipitated chromogenic substrate, the result can be evaluated by the naked eye.

As we have two mAbs specifically react to ζ-globin chains without cross-reactivity with other globin chains [19], these two mAbs were incorporated into the developed immunostick test for specifically detecting ζ-globin chains. Each stick is composed of four paddles (Fig. 1a). In the developed immunostick test, we therefore could include internal procedural controls which are positive control and negative controls (isotype-matched mAb control and uncoated control) within a single stick test (Fig. 1a). In the positive control, the paddle was coated with anti-mouse immunoglobulin antibody to capture the biotinylated mAb PL3, resulting in HRP-conjugated streptavidin binding. Consequently, this positive control might indicate the presence of biotinylated mAb PL3 in the reagent and the precise activity of HRP-conjugated streptavidin and precipitating chromogenic substrate. Negative controls, which included isotype-matched mAb control and uncoated paddle control, were used to differentiate non-specific background signal from the specific antigen-antibody signal [22–24]. As in sandwich immunoassays, rheumatoid factor, anti-animal IgG antibodies and heterophilic antibodies can lead to false positive results from the bridging of the capture and detecting antibodies in the system to yield a signal even in the absence of analyte [22–24]. This false positive is needed to be ruled out in any sandwich type immunoassay. In our developed immunostick test, the isotype-matched mAb control was, therefore, included. The isotype-matched mAb control was the paddle coated with irrelevant mouse IgG1 mAb. This control can determine the false positive due to the presence of rheumatoid factor, anti-animal IgG antibodies or heterophilic antibodies. Uncoated paddle control was also included in our developed immunostick test. This control was able to determine components in the sample other than rheumatoid factor, anti-animal IgG antibodies or heterophilic antibodies which could cause false positive signal. With these controls included in an individual immunostick test, this makes the developed immunostick test correctly determine the presence of ζ-globin chain in the sample.

The components in the samples may contribute to assay background [22–24]. It is necessary to determine the optimal dilution of the sample used. In this study, we found that hemolysate at dilution of 1:500 was the appropriate dilution for the developed immunostick test. Lesser dilutions exhibited assay background, which contributed to unreliability of result.

Generally, sandwich type immunoassay requires a complicated procedure and long time to run the assay (approximately 4 h). In order to reduce the assay procedure steps, we developed a simple immunostick test which combines the steps of ζ-globin chain capturing and detecting together. For this purpose, to avoid the high dose hook effect or postzone phenomenon, the titration of the appropriate concentration of biotinylated mAb PL3 (detecting mAb) and HRP-conjugated streptavidin for detection of ζ-globin chain in hemolysate (at dilution of 1:500) is obligatory [23]. Upon optimization, the simple immunostick test was successfully developed. The developed simple immunostick was proven to provide reliable results that could be obtained within 90 min.

The sensitivity and specificity of the developed immunostick test were determined in comparison to the gold standard PCR method. The developed immunostick test showed very good sensitivity (100%) and specificity (82%). Furthermore, the immunostick test has 100% NPV. By using the immunostick test for screening SEA type α-thalassemia 1, the negative results could exclude (−-(SEA)) α-thalassemia-1 trait. The PPV of immunostick was 76%, which was higher than the conventional screening protocol using mean corpuscular volume with cut-off 80 fl (approximately 40%) [25, 26]. Obviously, in usage for screening SEA type α-thalassemia 1, the developed immunostick test would significantly reduce cost and workload from DNA analysis to confirm screening results. As the pre-coated immunosticks were stable at room temperature for at least 20 weeks, commercialization of the assay is conceivable in the future. It is noted that within subjects carrying non-α thalassemia 1 gene, by the immunostick test, approximately 20% of these subjects showed positive reactivity. These results are similar to those obtained previously by using other immunoassay methods [17, 18]. The cause of this positivity, however, is still unknown. The cross-reactivity of anti-ζ-globin chain mAbs used with other globin chains have been assumed [18].

Currently, the ELISA test for detecting ζ-globin chain is commercially available. The price of the ELISA test is, however, very expensive which is around US$ 700–1000 per 96-microwell plate. This price is even higher when purchase from other countries than in USA. We then compared the cost of our established immunostick test and ELISA. The immunostick test is much less expensive. The approximate cost per test by ELISA and immunostick test are US$ 10 and 2, respectively.

In conclusion, we present here the development of a novel ζ-globin chain detection method: immunostick test. The developed method was a simple and reliable screening test to identify SEA type deletion α-thalassemia-1. By this technique, no sophisticated equipment is required and the result can be evaluated by the naked eye. The developed method, therefore, is valuable for screening α-thalassemia-1 in Southeast Asian countries where molecular testing is limited.

## Materials and Methods

### Antibodies

Mouse anti-ζ globin chain mAb clones PL2 (IgG1 isotype) and PL3 (IgG1) [19], mouse anti-Ag85B mAb clone AM85B-8B (IgG1, [27]) were generated in our laboratory. Goat anti-mouse immunoglobulins antibody was obtained from Jackson ImmunoResearch **(**West Grove, PA, USA). EZ-Link™ Sulfo-NHS-LC-Biotin was purchased from Pierce (Rockford, IL, USA). Horseradish peroxidase (HRP) and 3, 3′, 5, 5′-tetramethylbenzidine (TMB) substrate was purchased from Invitrogen (Camarillo, CA, USA). Precipitating TMB substrate was obtained from KPL (Gaithersburg, MD, USA). Nunc™ Immuno Stick was purchased from Thermo Fisher Scientific (MA, USA).

### Blood Samples

Ethical approval of this study was obtained from the Ethics Committee, Faculty of Associated Medical Sciences, Chiang Mai University (AMSEC-60EX-022).

The EDTA-anticoagulated blood specimens were submitted to Thalassemia Laboratory, the Associated Medical Sciences Clinical Service Center, Faculty of Associated Medical Sciences, Chiang Mai University and Thalassemia Research Center, Institute of Molecular Biosciences, Mahidol University, for hemoglobinopathy and thalassemia diagnosis. All samples were tested for α-thalassemia 1 SEA type deletions using real-time PCR and high-resolution melting analysis.

For determination of the concentration of ζ-globin chain in Hb Bart’s hydrops fetalis hemolysate, the concentration of total hemoglobin was measured by cyanmethemoglobinometry. Whole molecules of hemoglobin tetramer were separated by Acid-Urea-Triton X-100 polyacrylamide gel electrophoresis. Afterward, the globin chains were stained with Coomassie brilliant blue. Densitometry using an ImageScanner III (GE Healthcare) and the Image QuantT computer program were performed to determine the concentration of ζ-globin chain in hemolysate.

### Optimization of Capture mAb for Immunostick Test

Firstly, the anti-ζ globin chain mAb clone PL3 were biotinylated using EZ-Link™ Sulfo-NHS-LC-Biotin according to manufacturer instructions. Five μL of various concentrations of mAb PL2 or isotype-matched control mAb was dotted on each paddle of immunostick and incubated at 4 °C overnight. Sticks were washed with 0.05% Tween-PBS and blocked with 2% skimmed milk in PBS at 37 °C for 1 h. After washing, the sticks were dipped into hemolysate of Hb Bart’s hydrops fetalis containing 5 μg/mL of ζ-globin chain or normal subject. After incubation for 1 h at 37 °C, sticks were washed by dipping into 0.05% tween 20 in PBS (0.05% Tween-PBS) and briefly agitating for 5 s. Subsequently, sticks were dipped into 10 μg/mL of biotinylated mAb PL3. After washing, the antigen-antibody complex was monitored by immersing into HRP-labeled streptavidin at dilution of 1:5000. Sticks were washed and dipped into precipitating TMB substrate for 15 min. Blue color spot on the sticks, representing positive reaction, was observed.

### Determination of Optimal Dilution of Hemolysate

The 50 μg/mL of mAb PL2, 0.5 μg/mL of goat anti-mouse immunoglobulins antibody or 50 μg/mL isotype-matched control mAb were applied in a 5 μL dot on each paddle of immunostick and incubated at 4 °C overnight. Sticks were washed with 0.05% Tween-PBS and blocked with 2% skimmed milk in PBS at 37 °C for 1 h. After washing, the sticks were dipped into various dilutions of hemolysate of non-SEA type α-thalassemia 1. After incubation for 1 h at 37 °C, sticks were washed and dipped into 10 μg/mL of biotinylated mAb PL3. After washing, the antigen-antibody complex was monitored by immersing into HRP-conjugated streptavidin at dilution of 1:5000. Sticks were washed and dipped into precipitating TMB substrate for 15 min. Blue color spot on the sticks, representing positive reaction, was observed.

### Establishment of Simple Immunostick Test for Detection of ζ-Globin Chain

Each immunostick paddle was coated or uncoated with various mAbs as mentioned above and incubated at 4 °C overnight. Then, the coated immunosticks were blocked with 2% skimmed milk in PBS at 37 °C for 1 h. Immunosticks were dipped in the cocktail solution composed of ζ-globin chain containing hemolysate or normal hemolysate at dilution of 1:500, various concentrations of biotinylated mAb PL3 and HRP-conjugated streptavidin for 1 h at 37 °C. The immunosticks were washed by dipping in 0.05% tween 20 in PBS (0.05% Tween-PBS) and briefly agitating for 5 s. Subsequently, sticks were dipped into precipitating TMB substrate for 15 min. Blue color spot on the sticks, representing positive reaction, was observed.

### Validation of Immunostick Test for Screening of SEA Type Deletion α-Thalassemia 1 Carriers

The developed immunosticks were tested with 148 blood samples, including 95 non-α-thalassemia 1 SEA type deletion and 53 α-thalassemia 1 SEA type deletion. In comparison to the standard method, the sensitivity, specificity, PPV and NPV were determined.

### Stability of the Pre-Coated Immunostick Test

Each immunostick paddle was coated or uncoated with various mAbs as mentioned above and incubated at 4 °C overnight. Then, the coated immunosticks were blocked with 2% skimmed milk in PBS and incubated at 37 °C for 1 h. For long-term storage, pre-coated immunosticks were dipped into 10% glucose in PBS for 5 s. Subsequently, pre-coated immunosticks were dried and stored at room temperature for 20 weeks. The stored immunostick tests were tested for their stability on day 1, then weeks 1, 5, 10, 15 and 20 using hemolysate of SEA type α-thalassemia 1 and non-SEA type α-thalassemia 1.

## Data Availability

The datasets measured and analyzed during the study are available from the corresponding authors upon reasonable request.

## Abbreviations

ELISA: Enzyme-linked immunosorbent assay
HRP: Horseradish peroxidase
Ig: Immunoglobulin
mAb: monoclonal antibodies
PCR: Polymerase chain reaction
SEA type α-thalassemia 1: Southeast Asian type deletion α-thalassemia 1
